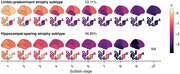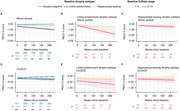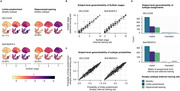# A generalizable data‐driven model of atrophy heterogeneity and progression in a memory clinic setting

**DOI:** 10.1002/alz.094033

**Published:** 2025-01-09

**Authors:** Hannah Baumeister, Jacob W. Vogel, Philip S. Insel, Luca Kleineidam, Steffen Wolfsgruber, Melina Stark, Helena M. Gellersen, Renat Yakupov, Matthias Schmid, Falk Lüsebrink, Frederic Brosseron, Gabriel Ziegler, Silka Dawn Freiesleben, Lukas Preis, Luisa Sophie Schneider, Eike Jakob Spruth, Slawek Altenstein, Andrea Lohse, Klaus Fliessbach, Ina R Vogt, Claudia Bartels, Björn H. Schott, Ayda Rostamzadeh, Wenzel Glanz, Enise I Incesoy, Michaela Butryn, Daniel Janowitz, Boris‐Stephan Rauchmann, Ingo Kilimann, Doreen Goerss, Matthias H. J. Munk, Stefan Hetzer, Peter Dechent, Michael Ewers, Klaus Scheffler, Anika Wuestefeld, Olof Strandberg, Danielle van Westen, Niklas Mattsson‐Carlgren, Shorena Janelidze, Erik Stomrud, Sebastian Palmqvist, Annika Spottke, Christoph Laske, Stefan Teipel, Robert Perneczky, Katharina Buerger, Anja Schneider, Josef Priller, Oliver Peters, Alfredo Ramirez, Jens Wiltfang, Michael T. Heneka, Michael Wagner, Emrah Düzel, Frank Jessen, Oskar Hansson, David Berron

**Affiliations:** ^1^ German Center for Neurodegenerative Diseases (DZNE), Magdeburg Germany; ^2^ Clinical Memory Research Unit, Lund University, Lund Sweden; ^3^ Department of Epidemiology & Biostatistics, University of California, San Francisco, San Francisco, CA USA; ^4^ Department of Neurodegenerative Diseases and Geriatric Psychiatry, University of Bonn Medical Center, Bonn Germany; ^5^ German Center for Neurodegenerative Diseases (DZNE), Bonn Germany; ^6^ Institute of Cognitive Neurology and Dementia Research (IKND), Otto‐von‐Guericke University, Magdeburg Germany; ^7^ Charité – Universitätsmedizin Berlin, corporate member of Freie Universität Berlin and Humboldt‐Universität zu Berlin, Berlin Germany; ^8^ Charité – Universitätsmedizin Berlin, corporate member of Freie Universität Berlin and Humboldt‐Universität zu Berlin – Institute of Psychiatry and Psychotherapy, Berlin Germany; ^9^ German Center for Neurodegenerative Diseases (DZNE), Berlin Germany; ^10^ Department of Psychiatry and Psychotherapy, Charité, Berlin Germany; ^11^ University Medical Center Goettingen (UMG), Goettingen Germany; ^12^ German Center for Neurodegenerative Diseases (DZNE), Goettingen Germany; ^13^ Faculty of Medicine and University Hospital Cologne, University of Cologne, Cologne Germany; ^14^ Institute for Stroke and Dementia Research (ISD), University Hospital, LMU, Munich Germany; ^15^ Sheffield Institute for Translational Neuroscience, University of Sheffield, Sheffield United Kingdom; ^16^ German Center for Neurodegenerative Diseases (DZNE), Rostock Germany; ^17^ Department of Psychiatry and Psychotherapy, University of Tuebingen, Tuebingen Germany; ^18^ MR‐Research in Neurosciences, Georg‐August‐University Goettingen, Germany, Goettingen Germany; ^19^ University of Tübingen, Tübingen Germany; ^20^ Imaging and Function, Skåne University Hospital, Lund Sweden; ^21^ Department of Neurology, Skåne University Hospital, Lund Sweden; ^22^ Memory Clinic, Skåne University Hospital, Malmö Sweden; ^23^ Department of Neurology, University of Bonn, Bonn Germany; ^24^ Section for Dementia Research, Hertie Institute for Clinical Brain Research and Department of Psychiatry and Psychotherapy, University of Tuebingen, Tuebingen Germany; ^25^ Department of Psychosomatic Medicine, Rostock University Medical Center, Rostock Germany; ^26^ LMU University Hospital, Munich Germany; ^27^ Division of Neurogenetics and Molecular Psychiatry, Department of Psychiatry and Psychotherapy, Faculty of Medicine and University Hospital Cologne, University of Cologne, Cologne Germany; ^28^ Luxembourg Centre for Systems Biomedicine (LCSB), University of Luxembourg, Luxembourg Luxembourg; ^29^ Center for Behavioral Brain Sciences (CBBS), Magdeburg Germany

## Abstract

**Background:**

Memory clinic patients are a heterogeneous population representing various aetiologies of pathological aging. It is unknown if divergent spatiotemporal progression patterns of brain atrophy, as previously described in Alzheimer’s disease (AD) patients, are prevalent and clinically meaningful in this group of older adults.

**Method:**

To uncover atrophy subtypes, we applied the Subtype and Stage Inference (SuStaIn) algorithm to structural MRI data from 813 participants (mean ± SD age = 70.67 ± 6.07 years, 52% females) from the DELCODE cohort. Participants were cognitively unimpaired (CU; n = 285) or patients with subjective cognitive decline (SCD; n = 342), mild cognitive impairment (MCI; n = 118), or dementia of the Alzheimer’s type (n = 68). Atrophy subtypes were compared in baseline demographics, fluid AD biomarkers, and domain‐specific cognitive performance. PACC‐5 trajectories over up to 240 weeks were examined. Clinical trajectories (PACC‐5 scores and MCI conversion rates) in only CU and SCD participants were analysed. SuStaIn modelling was repeated in participants from the Swedish BioFINDER‐2 study for replication and generalizability testing.

**Result:**

Limbic‐predominant and hippocampal‐sparing atrophy subtypes were identified (Figure 1). Limbic‐predominant atrophy first affected the medial temporal lobes, followed by further temporal and, finally, the remaining cortical regions. This subtype was related to older age, more pathological AD biomarkers, APOE e4 carriership, and an amnestic cognitive impairment. Hippocampal‐sparing atrophy initially occurred outside the temporal lobe and spared the medial temporal lobe until advanced stages. This atrophy pattern also affected individuals with positive AD biomarkers and was associated with more generalised cognitive impairment. Limbic‐predominant atrophy, in all and in only unimpaired participants, was linked to more negative longitudinal PACC‐5 slopes than observed in participants without or with hippocampal‐sparing atrophy (Figure 2) and increased the risk of MCI conversion. In BioFINDER‐2, analogous atrophy subtypes and cognitive correlates were identified. Group‐ and subject‐level model generalizability were excellent, indicating reliable performance in novel data (Figure 3).

**Conclusion:**

The proposed model is a promising tool for capturing heterogeneity among older adults at early at‐risk states for AD in applied settings. The implementation of atrophy subtype‐ and stage‐specific end‐points may increase the statistical power of pharmacological trials targeting early AD.